# Evaluating the Efficacy of Methocarbamol and Nefopam in Orthopedic Surgical Pain

**DOI:** 10.7759/cureus.59533

**Published:** 2024-05-02

**Authors:** Ahmed N Al-Nasrawi, Mustafa W Al-Ibrahim, Saifali J Aljabran

**Affiliations:** 1 Orthopaedic Surgery, Al-Sader Teaching Hospital, Basrah, IRQ; 2 Pharmacology, College of Medicine, University of Basrah, Basrah, IRQ

**Keywords:** side effects, post-operative analgesia, orthopedic operations, nefopam, methocarbamol

## Abstract

Introduction

Pain after orthopedic surgeries represents a special concern in patients with fractures. The use of multimodal analgesia significantly reduced the opioid need and reduced the risk of their side effects.

Objectives

This study compared the effectiveness and safety of methocarbamol and nefopam in the reduction of post-operative pain for patients undergoing orthopedic surgeries.

Method

This prospective, double-blind, randomized controlled trial took place at Al-Sader Teaching Hospital in Basrah, Iraq, from the first of February 2022 to the end of October 2023. The study aimed to assess the post-operative pain relief efficacy and safety of intramuscular nefopam (20 mg) and intravenous methocarbamol (1 g) in 110 adults (aged 18-65) undergoing elective orthopedic surgeries. Exclusions were made for allergies to the drugs, substance abuse history, and severe hepatic or renal impairment. Participants were randomized into two groups, with pain intensity measured at one hour, six hours, and 12 hours post-operation using the visual analog scale (VAS). Side effects were also evaluated. Statistical analysis was done using SPSS 27, with a significance level set at p<0.05 and a 95% confidence interval (CI).

Results

In this study, we conducted a rigorous comparison between two groups, methocarbamol and nefopam, to evaluate their efficacy and safety in post-operative pain management. We started by ensuring that the groups were well-matched in terms of age, gender distribution, and body mass index (BMI). The results showed remarkable similarities in mean age, gender distribution, and BMI, supported by robust p-values, affirming the effective matching of the two groups. Moving to pain management, we observed a significant advantage in favor of methocarbamol. At all-time intervals (one hour, six hours, and 12 hours post-operation), methocarbamol consistently demonstrated lower mean VAS scores compared to nefopam. These differences were highly statistically significant, underscoring the superior pain relief efficacy of methocarbamol. Exploring side effects, we found no statistically significant disparities in the occurrence of nausea and vomiting between the two groups. However, there was a noticeable trend toward higher tachycardia incidence in the nefopam group, though it did not reach statistical significance.

Conclusion

The present study showed a higher efficacy of methocarbamol in post-operative pain reduction in comparison to nefopam. No serious side effects were observed with both drugs.

## Introduction

Acute post-operative pain, which is frequently of medium-high degree, affects approximately 80% of patients who undergo surgery [1]. Less than half of surgical patients report receiving appropriate post-operative pain management. This percentage is a serious issue because it puts patients at an elevated risk of acquiring chronic pain related to the treatment and may result in unfavorable physiologic effects in the immediate post-operative term [2]. Adults with severe chronic post-operative pain account for 2% to 10% of cases [3].

The purpose of post-operative pain management is to ease the patient's return to normal activity and to lessen the harmful effects of acute post-surgical pain [4]. Opioid analgesic medication has always been the cornerstone of care for immediate post-operative pain. Nevertheless, the recent increase in morbidity and mortality linked to opioid abuse has increased the demand for more research into creating pain management plans that put a greater emphasis on employing a multimodal strategy [5]. Non-opioid analgesics with a range of modes of action offer the best pain treatment while lowering overall opioid intake and the adverse effects associated with opioid use. In numerous clinical studies, including orthopedic procedures, acetaminophen, gabapentin, lidocaine, ketamine, methocarbamol, dexmedetomidine, nefopam, and dexamethasone have shown opioid-sparing effects and the capacity to greatly reduce post-operative pain [6,7].

Nefopam is a centrally acting, non-opioid, and non-steroidal anti-inflammatory analgesic medication. Its mechanism of action for relieving pain involves the inhibition of sodium and calcium channels at the synaptic area of the dorsal horn of the spinal cord, leading to actions against nociceptive and neuropathic pain [8]. It was supposed to reduce morphine intake and post-operative pain [9]. Nefopam also has a stronger analgesic effect in hip arthroplasty patients with considerable pre-operative discomfort [10].

Methocarbamol is one of the centrally-acting striated skeletal muscle relaxants approved for the management of acute post-operative musculoskeletal pain and has been licensed since 1957 [11]. Despite being widely used today, there is a lack of many high-quality studies comparing methocarbamol to a placebo or other treatments for pain from the muscular origin, and there are no meta-analyses either [12]. However, off-label use has been studied for a variety of painful conditions, including acute and chronic non-specific low back pain, inflammatory arthritis, fibromyalgia, myofascial pain, rib fractures, and perioperative management of hip and knee replacements. Currently, clinical use is typically restricted to the adjunctive treatment of acute pain of musculoskeletal origin [13-15]. 

The goal of this study was to compare the effectiveness of methocarbamol and nefopam for treating post-operative pain following orthopedic surgery.

## Materials and methods

The study was conceived as a prospective, randomized, double-blind, and controlled trial aimed at assessing the post-operative analgesic efficacy and safety of nefopam and methocarbamol injections in orthopedic surgical patients. The investigation was conducted at the Orthopedic Department, Al-Sader Teaching Hospital, a reputable medical facility situated in Basrah, southern Iraq. It spanned from the first of February 2022 to the end of October 2023, providing a substantial timeframe to accrue, evaluate, and interpret the necessary data.

The cohort comprised 110 adults aged 18-65 years who were undergoing elective open orthopedic surgeries including lower limb trauma and hip replacement surgeries with ASA classification (1, 2, and 3) with surgical duration ranging from 1.5-2.5 hours. All patients were anesthetized with general anesthesia. In our study, we calculated the required sample size using the Steven K. Thompson formula, considering a population size of 550, a confidence level of 95%, and a population proportion of 10%. We aimed for a margin of error of 5%, which is critical for ensuring the reliability of our results within a specified confidence interval (CI). After applying these parameters in the formula, the calculated sample size was determined to be 110.

Exclusion criteria encompassed individuals with an allergy to methocarbamol or nefopam, a history of substance abuse, and severe hepatic or renal impairment. Participants were randomized using a computer-generated random number sequence into one of two treatment groups. Group A received a slow single dose of intravenous nefopam 20 mg over 15 minutes, and group B received methocarbamol infusion in a dosage of 1 g IV over five minutes as post-operative analgesics.

The primary outcome was the assessment of pain intensity at specified intervals post-operation. Secondary outcomes included evaluation of side effects associated with the administered drugs. Pain intensity was assessed at three post-operative intervals: one hour, six hours, and 12 hours using the visual analog scale (VAS). The evaluation of side effects was meticulously carried out, taking into account any adverse reactions or complications arising from the administration of nefopam and methocarbamol. Regarding patient comfort and ethical considerations, rescue analgesia was provided as necessary. Paracetamol 1 g intravenously was administered initially; if this proved insufficient, tramadol was used to ensure effective pain management throughout the study.

This study adhered to stringent ethical standards, having received approval from the Institutional Review Board of Basrah Health Directorate (No. 054786), and informed consent was signed by all participants before applying to the study. Data were collected using standardized data collection forms to capture demographic information, pain scores, adverse events, and other relevant data. Statistical analysis was performed using descriptive statistics to summarize demographic and clinical characteristics. Independent t-tests or Mann-Whitney U tests were used for continuous variables and Chi-square or Fisher’s exact tests for categorical variables. The significance level was set at a p-value of <0.05 with a CI of 95%.

## Results

Table 1 compares two groups, methocarbamol and nefopam, with respect to age, gender distribution, and body mass index (BMI). The mean ages are similar (46.36 vs. 47.24 years), and gender distribution is comparable with a slightly higher male percentage in both groups (69.1% vs. 63.6% for methocarbamol and nefopam, respectively). The mean BMIs also show no significant difference (34.38 vs. 35.18). These findings, supported by p-values of 0.485, 0.545, and 0.411, respectively, suggest that the two groups are well-matched in terms of these variables.

**Table 1:** Demographical data distribution among both groups BMI: body mass index

Variables		Methocarbamol group (No. 55)	Nefopam group (No. 55)	P-value
Age	Mean ± SD	46.36±8.62	47.24±7.24	0.485
Gender	Male	38 (69.1%)	35 (63.6%)	0.545
	Female	17 (30.9%)	20 (36.4%)	
BMI	Mean ± SD	34.38±5.68	35.18±6.52	0.411

In Table 2, a comparison is made between two groups, based on VAS at different time intervals. At one hour, the methocarbamol group had a significantly lower mean VAS score (3.58) compared to the nefopam group (5.31). This difference continued at six hours, with methocarbamol (4.82) showing lower pain scores than nefopam (5.53). After 12 hours, once again, methocarbamol (3.07) had lower pain scores compared to nefopam (4.71). All these differences were highly statistically significant with p-values <0.001, suggesting that methocarbamol was more effective in managing pain compared to nefopam at all time intervals.

**Table 2:** VAS analysis among both groups VAS: visual analog scale

VAS	Methocarbamol group (no. 55)	Nefopam group (no. 55)	P-value
At 1 hour (Mean ± SD)	3.58±0.629	5.31±1.06	<0.001
After 6 hours (Mean ± SD)	4.82±1.27	5.53±0.92	<0.001
After 12 hours (Mean ± SD)	3.07±0.92	4.71±1.18	<0.001

Table 3 compares side effects. There is no statistically significant difference in the occurrence of nausea and vomiting. Tachycardia shows a noticeable statistical significance, suggesting a potential trend toward higher tachycardia incidence in the nefopam group (21.81%) compared to the methocarbamol group (9.09%).

**Table 3:** Side effects analysis among both groups

Side effect	Methocarbamol group (no. 55)	Nefopam group (no. 55)	P-value
Nausea	1 (1.8%)	2 (3.6%)	0.628
Vomiting	0 (0.0%)	1 (1.8%)	0.315
Tachycardia	5 (9.09%)	12 (21.81%)	0.003

Table 4 compares vital signs in two groups treated with methocarbamol and nefopam. The analysis includes systolic and diastolic blood pressures and pulse rate at various time points post-administration. The results show no significant differences in systolic and diastolic blood pressures between the treatments across most time points, with all p-values exceeding the conventional significance level (0.05). However, there is a statistically significant difference in pulse rate at six hours (p-value of 0.031), indicating that the treatments affect heart rate differently over time.

**Table 4:** Vital signs analysis among both groups

Variables		Methocarbamol (no. 55)	Nefopam (no. 55)	P-value
Systolic blood pressure	At administration (Mean ± SD)	137.17±9.06	135.19±7.94	0.08
	After 1 hour (Mean ± SD)	135.24±7.96	133.56±7.31	0.06
	After 6 hours (Mean ± SD)	138.29±5.46	137.64±6.21	0.064
	After 12 hours (Mean ± SD)	129.0±6.82	127.41±5.22	0.057
Diastolic blood pressure	At administration (Mean ± SD)	83.96±6.40	85.56±8.22	0.421
	After 1 hour (Mean ± SD)	82.83±7.50	84.70±8.81	0.058
	After 6 hours (Mean ± SD)	79.45±4.67	80.66±6.14	0.069
	After 12 hours (Mean ± SD)	81.94±5.99	77.92±6.44	0.052
Pulse rate	At administration (Mean ± SD)	98.06±10.3	96.04±4.86	0.053
	After 1 hour (Mean ± SD)	105.74±6.58	110.22±6.30	0.058
	After 6 hours (Mean ± SD)	99.32±7.05	108.81±9.97	0.031
	After 12 hours (Mean ± SD)	97.82±7.81	97.08±8.17	0.485

## Discussion

As this study represents the inaugural investigation into the analgesic properties of methocarbamol and nefopam in alleviating post-operative pain, it contributes novel and substantial insights to the existing medical literature, which has advocated the utilization of multimodal analgesic approaches in the management of post-operative pain associated with fractures.

In recent years, there has been a discernible upsurge in the utilization of multimodal pain management strategies. These were aimed at enhancing pain control and mitigating opioid exposure. Methocarbamol, as a skeletal muscle relaxant, exerts its effects by inhibiting acetylcholinesterase activity at the neuromuscular junction, thus inducing muscle relaxation without a direct impact on striated muscle [16]. Interestingly, a retrospective investigation conducted by Aljuhani et al. failed to provide substantive evidence supporting the use of methocarbamol in the context of traumatic injury, as it did not demonstrate a significant reduction in pain intensity during hospitalization, opioid consumption, or duration of hospital stay [17]. Conversely, the current study reveals a significant difference, revealing a substantial discrepancy in pain alleviation between the methocarbamol and nefopam groups, particularly evident one hour post-administration. This observation aligns with the outcomes of a study conducted by Deloney et al., which explored the efficacy of methocarbamol in managing pain among patients with traumatic rib fractures and reported a marked reduction in pain scores [18].

Looke and Kluth (2013) conducted a retrospective cohort study at Florida Medical Center, comparing a new perioperative pain protocol involving pre-operative intravenous methocarbamol and acetaminophen to a 2008 protocol using oral analgesics. The study included 300 patients, split equally between the two protocols, and found significant reductions in opioid use, improvements in physical therapy outcomes, and shorter hospital stays for the current protocol group [15]. Contrasting with their study, our study investigates the effects of administering methocarbamol post-operatively rather than pre-operatively.

Nefopam administration both before the skin incision and upon the completion of surgery failed to exert a discernible influence on the total morphine consumption or the intensity of post-operative pain, as indicated by a study encompassing patients undergoing open spine procedures [19]. In a similar vein, an investigation involving patients subjected to minimally invasive spine surgery unveiled that the incorporation of a 24-hour nefopam infusion did not yield any incremental analgesic benefits or lead to improved functional outcomes post-surgery [20].

Possible explanations can be advanced to rationalize why nefopam demonstrated a comparatively weaker analgesic effect than methocarbamol in our study. Muscular spasms around the fractured bone or the surgical site significantly contributed to overall pain perception and limited range of motion, corroborating our findings [21]. This is particularly relevant given that a substantial proportion of the patients in our study presented with lower limb complaints, all of which are heavily encased by musculature, potentially intensifying the sensation of pain.

The safety profiles of both methocarbamol and nefopam were meticulously assessed in our study, and reassuringly, no severe adverse effects were observed with either medication. Nonetheless, it is noteworthy that tachycardia was more frequently documented among patients receiving nefopam compared to those administered methocarbamol. This aligns with prior research where approximately 50% of patients receiving nefopam experienced tachycardia in the immediate post-operative period [22]. It is imperative to acknowledge that various infrequent and non-serious adverse effects have been reported in association with methocarbamol use in previous studies, including drowsiness, skin rash, weakness, and hyperhidrosis [23]. Importantly, these side effects typically abate shortly after the cessation of treatment.

Table 5 below reviews several muscle relaxants, highlighting their use in multimodal analgesia along with notable contraindications and recommendations. Methocarbamol is distinguished for less sedation and dependency, recommended for muscle spasms and trauma [15,7]. Cyclobenzaprine and carisoprodol, both used for muscle spasms, carry risks like sedation and cardiac issues, or barbiturate-like effects, respectively [24,25]. Baclofen and tizanidine are advised for spasticity but come with serious potential withdrawal symptoms and hepatotoxicity [26,27]. Zolpidem mimics benzodiazepine effects, and ketamine is noted for its application in managing severe pain and opioid complications but with side effects including hypersalivation and hallucinations [28,29]. Local or regional anesthesia is flagged for complications like nerve injury and hypotension, primarily used in perioperative pain management [30]. The table effectively outlines the delicate balance between therapeutic benefits and potential risks associated with each medication, emphasizing the importance of tailored patient care based on specific health conditions and risk factors.

**Table 5:** Review studies of different muscle relaxants and their role in multimodal analgesia AE: adverse effect

Medication or class	Author	Recommended use	Contraindications and/or specific recommendations
Methocarbamol	Looke & Kluth 2013 [15], Patanwala et al. 2017 [7]	Muscle spasms, hip and knee replacements, trauma, and rib fractures	Renal impairment, methocarbamol is less sedating, has less psychological dependence potential, and has less significant withdrawal potential than other agents.
Cyclobenzaprine	Khwaja et al. 2010 [24]	Muscle spasms	AEs: cardiac arrhythmias, sedation, dizziness
Carisoprodol	Serfer et al. 2010 [25]	Muscle spasms	AEs: barbiturate-like effects
Baclofen	Pérez-Arredondo et al. 2016 [26]	Spasticity, spasticity with brain or spinal cord injuries	AEs: sedation, serious withdrawal (seizures), renal impairment
Tizanidine	Yazicioglu et al. 2016 [27]	Spasticity, muscle spasms	AEs: hepatotoxicity, withdrawal, renal impairment
Zolpidem	Hagan et al. 2020 [28]	Spasticity, muscle spasms	Benzodiazepines like effects
Ketamine	Patanwala et al. 2017 [29]	Severe post-operative pain, opioid tolerance, opioid-induced hyperalgesia, opioid-induced respiratory depression	AEs: hypersalivation, laryngospasm, hallucinations precautions; severe CV disease, pregnancy, active psychosis, elderly
Local and/or regional anesthesia	Choi et al. 2013 [30]	Perioperative pain management, rib or hip fracture	AEs: nerve injury, hypotension, hematoma relative contraindication; compartment syndrome, coagulopathy

## Conclusions

In conclusion, this study demonstrates the superior efficacy of methocarbamol in reducing post-operative pain when compared to nefopam. Furthermore, it is important to note that both drugs exhibited a favorable safety profile, with no reports of serious adverse effects.
